# Cardiac Rehabilitation Adherence and Long-Term Outcomes Post Myocardial Infarction: A Mixed-Methods Study of Patient Motivation, Barriers, and Program Satisfaction

**DOI:** 10.7759/cureus.113152

**Published:** 2026-07-22

**Authors:** Akshaya D Ramachandran, Keertana Shivamaru, Shilpa Deoke, Drashti D Vaddoriya, Mangala S Lalithamani, Mohit Agrawal

**Affiliations:** 1 Nephrology, Mahatma Gandhi Medical College and Research Institute, Pondicherry, IND; 2 Medicine, Sapthagiri Institute of Medical Sciences and Research Centre, Bengaluru, IND; 3 General Medicine, NKP Salve Institute of Medical Sciences and Research Centre and Lata Mangeshkar Hospital, Nagpur, IND; 4 Medicine, Gujarat Cancer Society (GCS) Medical College, Hospital, and Research Centre, Ahmedabad, IND; 5 Family Medicine, Mediora Smart Clinic, Kozhikode, IND; 6 Physical Medicine and Rehabilitation, SS Agrawal Institute of Physiotherapy and Medical Care Education, Navsari, IND

**Keywords:** adherence, barriers, cardiac rehabilitation, long-term outcomes, myocardial infarction, patient motivation, program satisfaction

## Abstract

Background

Participation in a cardiac rehabilitation (CR) program is a key component of secondary prevention following myocardial infarction (MI). However, adherence to CR varies considerably and may influence functional recovery and short-term clinical outcomes. Understanding the factors that facilitate or hinder participation may help optimize rehabilitation programs and improve patient outcomes. This prospective mixed-methods study assessed the association between adherence to CR and clinical outcomes four months after MI and explored the motivational factors, perceived barriers, and patient experiences influencing adherence.

Materials and methods

A prospective mixed-methods study was conducted among 150 post-MI patients who were followed for four months after enrolment. Participants were categorized according to CR adherence as high (>80% attendance; n = 87, 58%), moderate (50%-79%; n = 36, 24%), or low (<50%; n = 27, 18%). Quantitative outcomes included functional capacity assessed using the six-minute walk test (6MWT), medication adherence, recurrent myocardial infarction, and rehospitalization. Qualitative data were obtained through semi-structured interviews exploring perceived health benefits, barriers, motivators, and patient satisfaction. Quantitative data were analyzed using comparative statistical methods, while qualitative interviews underwent thematic content analysis.

Results

High adherence to CR was associated with significantly greater improvement in functional capacity (mean increase: 120 ± 25 m) compared with the moderate-adherence (65 ± 20 m) and low-adherence (30 ± 15 m) groups (p < 0.001). Recurrent myocardial infarction during the four-month follow-up occurred in 6% of participants in the high-adherence group compared with 19% and 30% in the moderate- and low-adherence groups, respectively (p < 0.05). Rehospitalization rates were also lower among participants with high adherence (10%) than among those with moderate (22%) or low adherence (37%) (p < 0.05). Qualitative analysis identified perceived health benefits, family support, and positive interactions with healthcare professionals as important facilitators of adherence, whereas financial constraints, occupational commitments, greater travel distance, transportation-related difficulties, and limited awareness of CR were the principal barriers. Higher satisfaction with the rehabilitation program was observed among participants with high adherence.

Conclusion

Higher adherence to CR was associated with improved short-term functional recovery and lower rates of recurrent myocardial infarction and rehospitalization during the four-month follow-up period. The mixed-methods findings indicate that both personal and health system factors influence adherence. Strategies that improve patient education, accessibility, family support, and patient-centered delivery of CR programs may help enhance adherence and optimize recovery following MI.

## Introduction

Despite advances in acute coronary interventions and pharmacotherapy, myocardial infarction (MI) remains one of the leading causes of mortality and cardiovascular morbidity worldwide. Cardiac rehabilitation (CR) is an internationally recommended component of secondary prevention and has been shown to improve survival, functional exercise capacity, and the quality of life among individuals recovering from MI [1,2]. Despite its established benefits, adherence to CR remains suboptimal worldwide, with fewer than 50% of eligible patients completing the recommended rehabilitation program in many settings. Poor adherence has been associated with increased rates of hospital readmission, recurrent ischemic events, and adverse clinical outcomes [3,4]. Multiple factors influence participation in CR, including patient motivation, psychological well-being, socioeconomic status, the accessibility of rehabilitation services, and perceptions regarding the benefits of rehabilitation [5].

Recent evidence suggests that behavioral determinants such as self-efficacy, illness perception, and family support play important roles in promoting sustained participation in CR. In addition, qualitative research has demonstrated that patients' experiences during rehabilitation, their perceived health benefits, and their satisfaction with program delivery substantially influence continued engagement and the completion of rehabilitation [6,7]. Quantitative studies have consistently reported that regular participation in CR is associated with lower mortality and improved adoption of healthy lifestyle behaviors [8]. Consequently, a mixed-methods approach that integrates objective clinical outcomes with patient experiences provides a more comprehensive understanding of the facilitators and barriers influencing adherence to CR. Such an approach may help identify practical strategies to improve participation, optimize patient-centered rehabilitation services, and ultimately enhance clinical outcomes following MI [9,10].

The present study aimed to investigate the association between adherence to CR and short-term clinical outcomes following MI. Specifically, it evaluated adherence levels, functional recovery, medication adherence, recurrent myocardial infarction, rehospitalization, and patient satisfaction while exploring the psychological, social, economic, and logistical factors influencing participation through a mixed-methods approach.

## Materials and methods

Study design and setting

This was a prospective observational mixed-methods study investigating the effect of adherence to cardiac rehabilitation on recovery following myocardial infarction (MI). The study was conducted over a period of four months, from 1 January 2026 to 30 April 2026, at a tertiary care hospital within an organized phase II cardiac rehabilitation program. Ethical approval was obtained from the Institutional Ethics Committee of the NKP Salve Institute of Medical Sciences and Research Centre and Lata Mangeshkar Hospital (approval number: NKPSIMS & RC and LMH/IEC/16/2026). Participants were recruited after the completion of phase I MI rehabilitation and were deemed medically fit for participation in supervised phase II cardiac rehabilitation. The study evaluated both quantitative clinical outcomes and qualitative aspects of patient behavior and experience.

Selection Criteria

Participants were included if they were aged ≥18 years, were diagnosed with MI according to standard clinical criteria, had completed phase I cardiac rehabilitation, were eligible for phase II rehabilitation, were hemodynamically stable, had received medical clearance for exercise-based rehabilitation, and provided written informed consent. Exclusion criteria included inability to perform physical activity due to musculoskeletal, neurological, or cognitive impairment; refusal to participate or inability to attend follow-up; terminal illness or poor prognosis; New York Heart Association (NYHA) class IV heart failure; or the presence of uncontrolled arrhythmias or medical instability.

Sampling and Sample Size

The sample size was calculated using the single population proportion formula, n = Z²P(1 − P)/d², where Z = 1.96, corresponding to a 95% confidence level; P = 0.50, a conservative estimate selected in the absence of reliable local prevalence data and supported by previous reports demonstrating wide variability in cardiac rehabilitation participation rates; and d = 0.08 (absolute precision) [10,11]. Based on these assumptions, the minimum required sample size was approximately 150 participants. Therefore, a total of 150 consecutive eligible patients were recruited during the study period. For the qualitative component, purposive sampling was employed to recruit participants representing all three medication adherence categories. Thirty participants (10 from each adherence group) underwent semi-structured interviews, which were considered sufficient to achieve thematic saturation.

Procedure

Eligible participants were enrolled following discharge from phase I cardiac rehabilitation, and baseline data were collected, including age, sex, ethnicity, the date of MI, medications, lipid profile, and ejection fraction. Functional capacity was assessed using the six-minute walk test (6MWT). Echocardiography was performed to measure left ventricular end-diastolic volume (LVEDV) and calculate ejection fraction. Adherence to cardiac rehabilitation was assessed through attendance records, medication logs, and program participation data. Medication logs documented the prescribed cardiovascular medications, daily medication intake as reported by participants, missed doses, and treatment interruptions. These records were reviewed during each scheduled follow-up visit and verified against prescription records whenever available. Attendance records included the number of scheduled and completed cardiac rehabilitation sessions, allowing the calculation of the overall adherence rate. Adherence was calculated as the percentage of scheduled cardiac rehabilitation sessions attended during the four-month follow-up. Participants attending >80% of scheduled sessions were classified as having high adherence, consistent with commonly accepted definitions of adherence in cardiac rehabilitation research [12]. Participants attending 50%-79% of scheduled sessions were categorized as having moderate adherence, whereas those attending <50% of scheduled sessions were considered to have low adherence. The latter categories were defined a priori by the investigators to differentiate clinically meaningful levels of program participation and facilitate comparative analyses across adherence groups.

The cardiac rehabilitation program consisted of supervised multidisciplinary sessions conducted three times per week over a four-month period. Each session lasted approximately 60 minutes and included aerobic exercise, resistance training, patient education, risk factor modification, dietary counseling, and medication adherence counseling, in accordance with institutional cardiac rehabilitation protocols [10].

Follow-up assessments were conducted four months after enrolment. Clinical outcomes, including recurrent myocardial infarction, unplanned hospitalization, and all-cause mortality, were recorded. Recurrent myocardial infarction was defined as a new myocardial infarction occurring during the four-month follow-up period after the index myocardial infarction. The primary outcome was improvement in functional capacity at four months as measured by the six-minute walk test (6MWT). Secondary outcomes included medication adherence, recurrent myocardial infarction, rehospitalization, and all-cause mortality. Qualitative interviews were conducted using a semi-structured interview proforma developed by the authors based on a review of the literature and expert consultation. The interview guide was pilot-tested for clarity and feasibility. Interviews were audio-recorded with participants' consent, transcribed verbatim, and analyzed using thematic content analysis.

Statistical analysis

Data were entered into Microsoft Excel (Microsoft Corp., Redmond, WA) and analyzed using SPSS version 26 (IBM Corp., Armonk, NY). Continuous variables were expressed as mean ± standard deviation (SD) and categorical variables as frequency (n) and percentage (%). The chi-square test (or Fisher's exact test, where appropriate) was used for categorical variables. One-way ANOVA (or Kruskal-Wallis test for nonparametric data) was used for continuous variables. Logistic regression analysis was performed to assess the association between adherence level and clinical outcomes at four months. A p-value of <0.05 was considered statistically significant.

## Results

A total of 150 post-myocardial infarction (MI) patients were included in the study. Overall, 87 (58%) patients demonstrated high adherence to the cardiac rehabilitation program, while 36 (24%) and 27 (18%) exhibited moderate and low adherence, respectively. Patients with high adherence showed significantly greater improvements in functional capacity, lipid profile, and medication compliance after four months compared with those with moderate or low adherence (p < 0.05). The proportion of patients who required rehospitalization for recurrent MI was lowest among those with high adherence (6%), compared with the moderate-adherence (19%) and low-adherence (31%) groups. Perceived health benefits, family encouragement, and positive interactions with therapists were the most commonly reported motivators for adherence, whereas financial constraints, occupational commitments, greater travel distance to the cardiac rehabilitation facility, transportation-related difficulties, and inadequate knowledge of the rehabilitation program were the principal barriers identified during the qualitative interviews.

Table 1 summarizes the baseline demographic characteristics of the study participants. There were no statistically significant differences in age or sex distribution among the high-, moderate-, and low-adherence groups (p > 0.05), indicating that the groups were comparable at baseline.

**Table 1:** Baseline characteristics of participants Values are expressed as mean ± standard deviation (SD) for continuous variables and number (percentage) for categorical variables. P-values were calculated using one-way ANOVA for continuous variables and the chi-square test for categorical variables

Variable	Total (N = 150)	High (n = 87)	Moderate (n = 36)	Low (n = 27)	P-value
Age (years), mean ± SD	56.4 ± 9.8	56.1 ± 8.9	57.5 ± 10.4	55.3 ± 9.7	0.62
Male, n (%)	102 (68.0)	57 (65.5)	26 (72.2)	19 (70.4)	0.73
Female, n (%)	48 (32.0)	30 (34.5)	10 (27.8)	8 (29.6)	

Table 2 shows that participants in the high-adherence group attended more than 80% of prescribed cardiac rehabilitation sessions, while the moderate-adherence group attended 50%-79% of sessions, and the low-adherence group attended less than 50%, demonstrating clear stratification in attendance patterns.

**Table 2:** Cardiac rehabilitation attendance pattern Adherence categories were defined based on the percentage of prescribed cardiac rehabilitation sessions attended

Adherence group	Participants, n (%)	Attendance definition
High adherence	87 (58%)	>80% sessions attended
Moderate adherence	36 (24%)	50%-79% sessions attended
Low adherence	27 (18%)	<50% sessions attended

Table 3 shows that adverse clinical outcomes during the four-month follow-up were lowest in the high-adherence group, whereas recurrent myocardial infarction occurring after the index myocardial infarction and subsequent rehospitalization were more frequently observed among patients with moderate and low adherence.

**Table 3:** Clinical outcome comparison at four months Values are expressed as numbers (percentages). P-values were calculated using the chi-square test or Fisher's exact test, where appropriate MI: myocardial infarction

Outcome	High (n = 87)	Moderate (n = 36)	Low (n = 27)	P-value
Recurrent MI	5 (6%)	7 (19%)	8 (30%)	0.01
Rehospitalization	9 (10%)	8 (22%)	10 (37%)	0.02

Table 4 shows that improvement in functional capacity, as measured by the six-minute walk test, was greatest in the high-adherence group, moderate in the intermediate group, and minimal in the low-adherence group.

**Table 4:** Functional capacity (6MWT improvement) Values are expressed as mean ± standard deviation (SD) 6MWT: six-minute walk test

Group	Mean improvement (meters) ± SD
High	120 ± 25
Moderate	65 ± 20
Low	30 ± 15

Table 5 shows that perceived health benefits, family support, and positive program or staff experience were the most frequently reported motivational factors influencing participation in cardiac rehabilitation.

**Table 5:** Motivational factors identified from qualitative interviews (N = 30) Frequencies represent the number and percentage of interview participants (N = 30) reporting each theme. Multiple responses per participant were permitted

Theme	Frequency, n (%)
Health benefit perception	22 (73%)
Family support	18 (60%)
Program experience/staff support	16 (53%)

Table 6 shows that financial constraints, work-related commitments, and low awareness or motivation were the most reported barriers among participants with low adherence.

**Table 6:** Barriers identified from qualitative interviews (N = 30) Frequencies represent the number and percentage of interview participants (N = 30) reporting each theme. Multiple responses per participant were permitted

Barrier	Frequency, n (%)
Financial constraints	14 (47%)
Work commitments	13 (43%)
Low awareness/motivation	12 (40%)

The qualitative analysis of the semi-structured interviews identified four major themes influencing adherence to the cardiac rehabilitation program: perceived health benefits, family and social support, financial and logistical barriers, and knowledge and motivation regarding cardiac rehabilitation. Interview transcripts were independently coded by two investigators using thematic content analysis, and discrepancies were resolved through discussion until consensus was achieved. Representative participant quotations illustrating each theme are presented in Table 7.

**Table 7:** Summary of qualitative themes influencing adherence to cardiac rehabilitation

Theme	Description	Representative participant quotation
Perceived health benefits	Participants reported that participation in cardiac rehabilitation improved their physical fitness, confidence, and recovery after myocardial infarction, motivating continued attendance	"The rehabilitation sessions helped me regain my strength. I could walk farther and felt much more confident after a few weeks"
Family and social support	Encouragement from family members and positive interactions with healthcare professionals motivated participants to continue attending scheduled sessions	"My family encouraged me to attend every session, and the therapists always motivated me to keep going"
Financial and logistical barriers	Financial constraints, occupational responsibilities, greater travel distance to the rehabilitation center, and transportation difficulties limited regular attendance	"Travelling to the hospital three times a week was difficult because it was far from my home and transportation was expensive"
Knowledge and motivation	Participants with a better understanding of the benefits of cardiac rehabilitation were more motivated to adhere, whereas inadequate awareness reduced participation	"Once I understood how rehabilitation could prevent another heart attack, I made sure not to miss my sessions"

The qualitative findings complemented the quantitative results by providing a deeper understanding of the facilitators and barriers influencing adherence to cardiac rehabilitation. Participants who perceived clear health benefits and received family support demonstrated greater adherence, whereas financial constraints, occupational commitments, travel distance, transportation difficulties, and limited awareness of the rehabilitation program emerged as important barriers to regular participation.

## Discussion

The present prospective mixed-methods study demonstrated that higher adherence to a cardiac rehabilitation (CR) program following myocardial infarction (MI) was associated with more favorable short-term clinical and functional outcomes. Adherence to CR is a cornerstone of secondary prevention and has consistently been associated with improved cardiovascular outcomes and quality of life [2]. In the present study, 58% of participants attended more than 80% of their scheduled CR sessions, which is comparable to previous reports demonstrating that completion rates of CR programs remain suboptimal worldwide [11]. Our findings showed that participants with high adherence demonstrated significantly greater improvements in functional exercise capacity and medication adherence, together with lower rates of recurrent myocardial infarction and rehospitalization during the four-month follow-up, compared with those exhibiting moderate or low adherence.

These findings are consistent with previous studies demonstrating that participation in CR programs reduces cardiovascular morbidity through structured exercise, risk factor modification, and the optimization of secondary prevention strategies [4]. Similarly, patients who maintained higher adherence experienced fewer recurrent cardiovascular events and hospital readmissions, supporting previous evidence that sustained participation in CR is associated with improved clinical outcomes [8]. Importantly, our findings suggest that continued participation in rehabilitation may be more influential than enrolment alone. However, given the observational design of the present study, these findings should be interpreted as associations rather than evidence of causality. The qualitative component complemented these observations by providing additional insight into the factors influencing adherence beyond attendance records alone.

Qualitative analysis identified four major themes influencing adherence: perceived health benefits, family and social support, financial and logistical barriers, and knowledge and motivation regarding CR. Participants who perceived improvements in their health and received encouragement from family members and healthcare professionals were more likely to remain engaged with rehabilitation. These observations are consistent with previous literature demonstrating that social support and positive patient-provider relationships are important determinants of adherence [5]. Conversely, financial constraints, occupational commitments, greater travel distance to the CR facility, transportation-related difficulties, and inadequate awareness of the rehabilitation program emerged as major barriers to sustained participation, supporting previous reports identifying socioeconomic and accessibility challenges as significant obstacles to CR attendance [13]. The qualitative findings therefore complemented the quantitative results by providing a better understanding of the psychological, social, and environmental factors influencing patient participation, reinforcing recommendations advocating patient-centered rehabilitation models that address individual patient needs [7].

Patient satisfaction was positively associated with adherence, suggesting that program quality, accessibility, and effective communication between healthcare professionals and patients play important roles in maintaining long-term engagement [14]. These findings support previous recommendations advocating flexible scheduling, home-based or tele-rehabilitation models, individualized counselling, and family involvement as strategies to improve participation and reduce dropout rates [10,15].

By integrating quantitative clinical outcomes with qualitative patient experiences, this study provides a more comprehensive understanding of adherence to CR following MI [16]. The findings suggest that strengthening patient education, improving accessibility, providing financial and family support, and fostering a supportive therapeutic environment may help improve adherence and ultimately enhance clinical outcomes [17]. Future multicenter studies with larger sample sizes and longer follow-up periods should evaluate interventions specifically designed to overcome the identified barriers and determine whether improved adherence results in sustained long-term cardiovascular benefits [18].

Strengths and limitations

The present study has several strengths. Its prospective mixed-methods design enabled the integration of quantitative clinical outcomes with qualitative patient experiences, providing a comprehensive understanding of factors influencing adherence to CR following MI. The inclusion of both objective clinical outcomes and patient-reported experiences enhances the clinical relevance of the findings and provides practical insights into the facilitators and barriers affecting participation in rehabilitation. Furthermore, the prospective design and standardized assessment of functional outcomes strengthen the reliability of the study findings.

This study also has several limitations. First, it was conducted at a single tertiary care center, which may limit the generalizability of the findings to other healthcare settings and populations. Second, medication adherence and patient satisfaction were assessed using self-reported data, introducing the possibility of recall and response bias. Third, the four-month follow-up period may not adequately capture long-term adherence patterns or sustained cardiovascular outcomes. Fourth, the observational study design precludes causal inference, and residual confounding cannot be excluded. Finally, although qualitative interviews were independently analyzed using thematic content analysis, the interpretation of participant narratives remains inherently subjective despite efforts to enhance analytical rigor.

## Conclusions

Higher adherence to cardiac rehabilitation following myocardial infarction was associated with improved short-term functional recovery, lower rates of recurrent myocardial infarction, and fewer rehospitalizations during the four-month follow-up period. The mixed-methods approach demonstrated that adherence was influenced by multiple interrelated factors, including perceived health benefits, family support, motivation, and positive experiences with the rehabilitation program, whereas financial constraints, occupational commitments, travel distance, transportation-related difficulties, and limited awareness of cardiac rehabilitation emerged as important barriers to participation. These findings highlight the importance of patient-centered strategies that improve education, accessibility, and support systems to enhance adherence and optimize recovery following myocardial infarction.

## Appendices

Table 8 shows the semi-structured interview proforma.

**Table 8:** Semi-structured interview proforma

Question number	Semi-structured interview question
1	Can you describe your experience participating in the cardiac rehabilitation program?
2	What motivated you to attend cardiac rehabilitation sessions regularly?
3	What benefits, if any, have you noticed since participating in cardiac rehabilitation?
4	What challenges or barriers made it difficult to attend rehabilitation sessions?
5	Did family members or friends influence your participation in the program? If yes, how?
6	How would you describe your interactions with the healthcare professionals involved in the rehabilitation program?
7	Were there any financial, transportation, or work-related difficulties that affected your attendance?
8	How satisfied were you with the rehabilitation program overall?
9	What changes would you suggest to improve cardiac rehabilitation services?
10	Is there anything else you would like to share regarding your recovery after myocardial infarction?
